# *Rhizoctonia solani* Kühn Pathophysiology: Status and Prospects of Sheath Blight Disease Management in Rice

**DOI:** 10.3389/fpls.2022.881116

**Published:** 2022-05-03

**Authors:** Manoranjan Senapati, Ajit Tiwari, Neha Sharma, Priya Chandra, Bishnu Maya Bashyal, Ranjith Kumar Ellur, Prolay Kumar Bhowmick, Haritha Bollinedi, K. K. Vinod, Ashok Kumar Singh, S. Gopala Krishnan

**Affiliations:** ^1^Division of Genetics, ICAR-Indian Agricultural Research Institute, New Delhi, India; ^2^Division of Plant Pathology, ICAR-Indian Agricultural Research Institute, New Delhi, India

**Keywords:** *Rhizoctonia solani*, rice sheath blight (ShB), biological control, disease resistance, transgenic rice, resistance QTLs

## Abstract

Sheath blight caused by necrotrophic fungus *Rhizoctonia solani* Kühn is one of the most serious diseases of rice. Use of high yielding semi dwarf cultivars with dense planting and high dose of nitrogenous fertilizers accentuates the incidence of sheath blight in rice. Its diverse host range and ability to remain dormant under unfavorable conditions make the pathogen more difficult to manage. As there are no sources of complete resistance, management through chemical control has been the most adopted method for sheath blight management. In this review, we provide an up-to-date comprehensive description of host-pathogen interactions, various control measures such as cultural, chemical, and biological as well as utilizing host plant resistance. The section on utilizing host plant resistance includes identification of resistant sources, mapping QTLs and their validation, identification of candidate gene(s) and their introgression through marker-assisted selection. Advances and prospects of sheath blight management through biotechnological approaches such as overexpression of genes and gene silencing for transgenic development against *R. solani* are also discussed.

## Introduction

Rice (*Oryza sativa L*.) serves as the primary diet for approximately 67% of the world population. In the Asian region, the demand for rice production is the highest in the world, due to the increased preference for rice among the population (Mohanty, 2013). Throughout the world, productivity of rice is affected by several biotic and abiotic factors. There are about 50 different biotic factors that can cause potential yield loss in rice including fungi, bacteria, viruses, nematodes and insects. Of the disease-causing organisms, fungal pathogens impose a greater challenge in sustaining rice production (Webster and Gunnell, 1992).

Among the fungal diseases causing significant yield loss in rice, sheath blight is ranked the second most important after rice blast (Pan et al., 1999). The sheath blight pathogen has two stages, *Rhizoctonia solani* Kühn, the anamorph stage and a teleomorph stage, *Thanatephorus cucumeris* (Frank) Donk. Belonging to the division Basidiomycota, *R. solani* is a necrotrophic fungus that produces sclerotia of varying sizes but with uniform texture, which can remain dormant for many years (Mukherjee, 1978). The disease causes a yield reduction ranging from 20 to 50% depending on the severity of infection (Groth and Bond, 2007; Margani and Widadi, 2018). In the recent past, sheath blight has become a major threat, especially under intensive rice cultivation. Monoculture of high-yielding semi-dwarf rice varieties, heavy doses of nitrogenous fertilizers and the favorable micro-environment facilitated by the crop density are implicated as the major factors favouring the sharp increase in the disease incidence (Savary et al., 1995; Cu et al., 1996). Reported for the first time in Japan in 1910 (Miyake, 1910), sheath blight disease had spread all across the world. *R. solani* is a very destructive pathogen. Taking advantage of the large host range (Kozaka, 1965), the pathogen often survives on the alternate hosts during hostile conditions, making the disease very difficult to manage. Besides, it can also survive in soil and dead plant debris by producing resting structures such as sclerotia.

To incite the disease in rice plants, the fungal inoculum should come in contact with the live host tissues in the field. The inoculum can be a runner hypha or a sclerotium and in rare cases basidiospores, often floating in the irrigation water. By this mode, inoculum can travel and spread to different locations in the field or from the irrigation canals where alternate hosts can supply sufficient inoculum. In rice, *R. solani* can infect the plant at any growth stage (Dath, 1990). The incidence of sheath blight is more severe in early maturing, semi-dwarf, highly tillering and compact cultivars (Bhunkal et al., 2015b). The disease severity and incidence increase with plant age (Singh et al., 2004). The resistance and susceptibility in the rice genotypes are distinct in mature plants as compared to seedlings (Dath, 1990). The sheath blight progression is slow in initial growth stages, while it is fast at tillering and later stages of growth (Thind et al., 2008).

Although several cultural, chemical and biological control strategies have been suggested to manage sheath blight disease of rice (Yellareddygari et al., 2014; Datta and Vurukonda, 2017), chemical control has been the most widely used method so far. However, this method is relatively less sustainable in crop production because of the increased cost of production, development of fungicide tolerance and apprehensions of residual toxicity. Biological strategies targeting host plant resistance have been advocated as the most viable solution, which includes mapping of gene(s) or quantitative trait loci (QTLs) governing disease resistance and introgression to elite cultivars through molecular breeding. Additionally, novel biotechnological approaches like RNAi, transgenics and genome-editing approaches can also be used to generate a new resistance spectrum against *R. solani*. There are several reviews made previously on the sheath blight tolerance in rice, but most of which provide relatively less focus on breeding for resistance. In the present review, we have made a comprehensive update on the understanding of the pathophysiology of *R. solani* keeping in view crop varietal improvement and biological management of the sheath blight disease in rice. The review also summarizes a critical analysis of the pathogen diversity, host range, pathogenicity and genetics of rice plant resistance. Various approaches adopted in managing the disease through development of resistant varieties have also been described including the novel biotechnological approaches.

## Diversity of *R. Solani*

### Morphological Diversity Based on Anastomosis of Vegetative Hyphae

Anastomosis is a key process for a large number of filamentous fungi that facilitates the fusion of cell walls, cytoplasm and nucleus between genetically similar groups. An anastomosis group (AG) is a collection of closely related isolates grouped based on the ability of vegetative hyphae to anastomose/fuse with one another (Parmeter et al., 1969). *R. solani* is classified into different AGs based on their hyphal capability to fuse with tester hyphal mycelium (Carling, 1996; Craven et al., 2008). The fungus is assigned with fourteen different AGs starting from AG1 to AG13 and AGB1 as a bridging group. The 14 AGs exhibit wide variation in morphology of mycelial colony, nutritional requirement, host range and pathogenic virulence (Carling et al., 2002a,b; Ajayi-Oyetunde and Bradley, 2018). The anastomosis grouping of *R. solani* causing sheath blight of rice indicated that it belonged to AG1 group. Further grouping of AGs into different intraspecific subgroups (ISGs) have been carried out based on their DNA sequence and its homology, colony morphology, pathogenicity, isozyme pattern, rDNA-internal transcribed sequences and fatty acid composition. Classification of AG1 resulted in three subgroups, AG1-IA, AG1-IB, and AG1-IC, all causing blight (Ogoshi, 1987; Sneh et al., 1991; Carling, 1996). Among these, majority of the rice sheath blight pathogen belongs to the AG1-IA subgroup.

## Genetic Variability in *R. Solani*

Considerable morphological, pathogenic and genetic diversity has been established within *R. solani* isolates obtained from different parts of the world (Shu et al., 2014; Yugander et al., 2015). Taheri et al. (2007) could group a set of 150 isolates of *R. solani* collected from different parts of India into 33 groups at an 80% genetic similarity level using amplified fragment length polymorphism markers. Twenty-nine isolates from Bangladesh were grouped into two clusters by Ali et al. (2004) while Moni et al. (2016) grouped 18 isolates into four clusters. However, there was no significant correlation between virulence variation and genetic groups identified based on random amplified polymorphic DNA (RAPD) markers (Yi et al., 2002). In China, 175 isolates of *R. solani* belonging to AG1-IA showed considerable variability in virulence (Wang et al., 2015c). They could classify the isolates into weakly virulent, moderately virulent and highly virulent classes based on disease severity, which represented 28.0, 63.4 and 8.6% of isolates, respectively. Further establishing the genetic variability, as many as 80 alleles were detected using RAPD markers from 25 *R. solani* isolates collected from different geographic regions of India (Singh et al., 2015). The number of alleles per locus varied from 1 to 7.

Initially, the genome size of *R. solani* was estimated to be between 36.9 and 42.5 Mb with 11 chromosomes ranging in size from 0.6 to 6 Mb (Keijer, 1996). Later, a draft genome sequence of *R. solani* AG1-IA strain with a size of 36.94 Mb was released using next-generation sequencing technology (Zheng et al., 2013). Subsequently, another draft genome sequence of *R. solani* AG1-IA strain, 1802/KB (GenBank accession number KF312465) isolated from a popular rice variety from Malaysia, was generated with a size of 28.92 Mb (Nadarajah et al., 2017). Besides, a web-based database, RSIADB was constructed using the genome sequence (10489 genes) and annotation information for *R. solani* AG1-1A to analyze its draft genome and transcriptome (Chen et al., 2016).

### Host Range

*Rhizoctonia solani* is pathogenic against a diverse range of about 250 host plant species belonging to members of Poaceae, Fabaceae, Solanaceae, Amaranthaceae, Brassicaceae, Rubiaceae, Malvaceae, Asteraceae, Araceae, Moraceae, and Linaceae (Chahal et al., 2003). As many as 188 plant species belonging to 32 families were found to be infected by this fungus in Japan (Kozaka, 1961). Tsai (1974) reported *R. solani* infection in 20 species of 11 families in Taiwan, while it was found to infect 10 types of grasses and a *Cyperus* spp. in Thailand (Dath, 1990). In India, it has been reported on 62 economically important plants and 20 families of weeds (Roy, 1993). Several weed plant species have been identified to act as collateral hosts for the pathogen in absence of rice plants (Acharya and Sengupta, 1998), and serve as inoculum and aid in further spread of the disease (Kannaiyan and Prasad, 1980; Srinivas et al., 2014).

### Disease Symptoms

**FIGURE 1:**
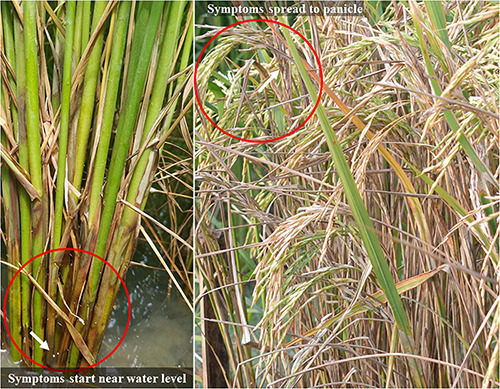
Symptom of sheath blight disease in rice; left side shows the initial symptoms appear on leaf sheath starting from water level, and the right side shows the disease spread up to panicle.

On infection, the fungus causes a range of symptoms including sheath blight, foliar blight, leaf blight, web-blight, head rot, bottom rot and brown patch in different crops. In rice, *R. solani* mainly attacks the leaf sheath and leaf blades and in severe cases, the whole plant including the emerging panicles may be affected (Rangaswami and Mahadevan, 1998). The disease symptoms on the infected plant can be visualized within 24–72 h after infection depending on the environmental conditions. Although the disease can occur at any growth phase, rice crop is most vulnerable at the tillering phase (Singh et al., 1988). Fungal mycelium determines the size and shape of lesions which are produced in patches of varying sizes (Ou et al., 1973). The typical symptom (Figure 1) is the appearance of greenish-gray water-soaked lesions on the leaf sheath near the water level that are circular, oblong or ellipsoid and about 1 cm long. These lesions enlarge and attain irregular shape, the center of which becomes gray white with brown margins. Lesions may appear on any part of the sheath and several lesions may coalesce to encircle the whole stem. Under favorable conditions, the infection may spread to upper leaf sheaths and leaf blades, which ultimately results in the rotting of leaf sheath and drying up of the whole leaf. In severe cases, the infection spreads to the panicle affecting grain filling and leading to the discoloration of seeds with brownish-black spots or black to ashy gray patches (Singh et al., 2016). In acute cases, the disease causes the death of the whole leaf, tiller and even the whole plant. At the field level, the infection usually affects the plants in a circular pattern referred to as ‘bird’s nest’ (Hollier et al., 2009).

### The Disease Cycle

**FIGURE 2:**
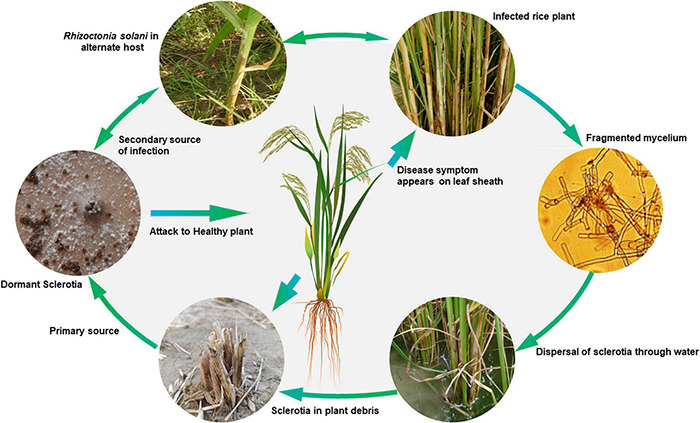
Disease cycle of sheath blight of rice caused by *Rhizoctonia solani* AG1-IA.

*Rhizoctonia solani* is a seed- and soil-borne pathogen, which survives through sclerotia and mycelia in infected seeds or soil in tropical environments. In soil, infected plant debris is the major carrier that may arise from rice or weed hosts (Figure 2). In temperate regions, soil and crop residue borne sclerotia act as the primary source of inoculum, which can spread through irrigation water from one field to another (Kozaka, 1970). Under favorable conditions, the sclerotia germinate to form mycelia, which on establishing contact with the rice plant surface grows and produces infection structures such as infection cushions and lobate appressoria. These infection structures aid mycelial penetration into the plant tissues. However, in some cases, infection occurs through stomata, where no infection structures are observed (Marshall and Rush, 1980). The pathogen spreads both vertically and horizontally with a horizontal spread of up to 20 cm/day under field conditions is reported (Savary et al., 1995). Plant to plant and field to field spread of the disease takes place through floating sclerotia and mycelia dispersed through rainfall and irrigation water runoff. Infected seeds are the primary source of inoculum for the spread of this disease to new areas. The seed infection and transmission of the pathogen from seed to seedlings in the form of lesions varies from 4.6–14.0% under field conditions (Sivalingam et al., 2006). Wind also helps in the secondary spread of the disease by dispersing the basidiospores to new fields. The basidia hymenium acts as a continuous source of secondary inoculum.

### GEOGRAPHICAL DISTRIBUTION OF *R. SOLANI*

**FIGURE 3:**
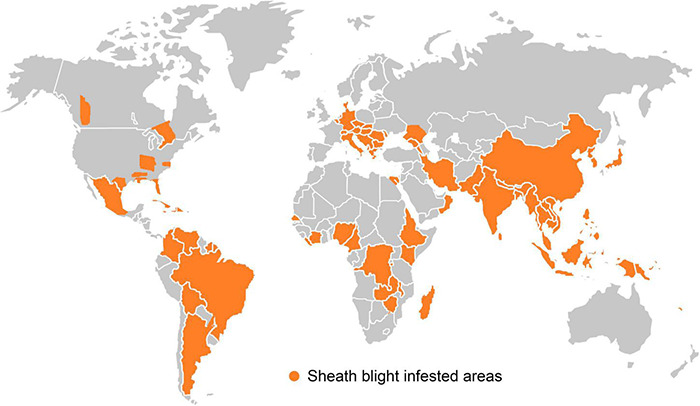
Geographical distribution of sheath blight disease occurrence in different countries of the world.

Since its first report in Japan in 1910, the pathogen has spread to most of all the rice growing areas in the world (Figure 3). This disease is recognized as a serious problem in the top ten rice growing countries *viz*. China, India, Indonesia, Bangladesh, Vietnam, Thailand, Burma, Philippines, Pakistan and Brazil (Singh et al., 2016). Incidence of sheath blight disease of rice in India was reported for the first time from Gurdaspur in Punjab (Paracer and Chahal, 1963). Later on, the disease has become a major problem in rice producing areas of eastern Uttar Pradesh, Uttarakhand, Bihar, West Bengal, Haryana, Odisha, Chhattisgarh, Tamil Nadu, Kerala, Karnataka, Andhra Pradesh, Jammu and Kashmir, Madhya Pradesh, Assam, Tripura and Manipur. The disease incidence was particularly severe among the high yielding semi-dwarf rice varieties, owing to their narrow genetic base, high dependency on chemical fertilizers and favorable weather. Due to the widespread incidence, economic losses to the tune of up to 58% in rice yield have been reported (Chahal et al., 2003).

### Pre-disposing Factors Affecting the Epidemiology

High ambient air temperature in combination with high relative humidity in the forenoon and wet leaves are major predisposing factors for sheath blight development in rice (Castilla et al., 1996; Biswas et al., 2011). Favorable temperature and evaporation rate results in 23.0 and 61.1% of disease incidence under field conditions, respectively (Lenka et al., 2008). The maximum progression of the disease is observed at the temperature range of 25°–30°C and relative humidity of 80–100% (Thind et al., 2008; Bhunkal et al., 2015a). The disease severity and yield loss increase with excess nitrogen application (Tang et al., 2007), and are accentuated in the presence of brown plant hopper and rice root-knot nematode, *Hirschmaniella oryzae* (Dath, 1990) and rice tungro virus (Sarkar and Chowdhury, 2007). Another factor under which severe incidence is seen is when the crop canopy is dense with high contact frequency between tissues (Huang et al., 2007). There is also a difference seen between the disease incidence among two sub-species of rice, *indica* and *japonica*, with the former having relatively higher tolerance than *japonica*. However, Lee and Rush (1983) reported that *japonica* cultivars with short and medium grains have higher resistance than long grain *indica* rice cultivar from the southern United States. Indicating the importance of nitrogen, Dath (1990) found a reduction in disease severity with the use of slow-release nitrogenous fertilizer such as Crotonylidene diurea (CDU) and Guanyl urea phosphate with the solo application of silica, phosphorus and potash. Increased dose of nitrogen and phosphorus reduces the incubation period as well as phenolic contents, leading to high disease severity, while application of K, Zn, S, and Fe reduce disease severity (Prasad et al., 2010). Application of soil amendments including neem cake, farm yard manure (FYM), vermicompost and rice husk (Senapoty, 2010) and spraying Ganoderma diethyl ester formulation (Sajeena et al., 2008) can reduce the disease incidence. Long-term field experiments revealed that *R. solani* sclerotia population and sheath blight disease severity remained low in conventional seeded plots as compared to stale seedbeds and no-till seedbeds (Cartwright et al., 1997). Minimal tillage also promotes sheath blight development (Rodriguez et al., 1999). Besides, the rate of infection was less in direct-seeded rice than in transplanted rice irrespective of spacing. Certain crop cycles can also influence the disease incidence pattern as seen with soybean in rotation with rice which leads to a heavy incidence of sheath blight (Rodriguez et al., 2003; Groth and Bond, 2007).

### Host-Pathogen Interaction Between Rice and *R. solani*

To colonize and establish the disease in rice plants, *R. solani* employs a variety of tactics. Effector proteins are used by pathogens to infect the host plant and cause disease. *R. solani* is known to produce several effector molecules (Table 1) with varying functions enabling successful colonization. The primary requirement for *R. solani* infection is the degradation of the plant cell wall. *R. solani* AG1-IA is predicted to produce as many as 223 carbohydrate-active enzymes (CAZymes) such as glycoside hydrolases, glucosyltransferases, and polysaccharide lyases (Zheng et al., 2013). Polygalacturonase hydrolyses the pectin in the plant cell wall, which results in cell death (Chen et al., 2017). During the infection process, the pathogen secretes oxalate and transgenic rice plants overexpressing oxalate oxidase break oxalate and enhance resistance against sheath blight (Molla et al., 2013). *R. solani* has also been reported to use α-1,3-glucans to mask the chitin on its surface and evade the host defense mechanism (Fujikawa et al., 2012). When an extracellular signal is received, the fungi activate different signal transduction pathways for pathogenicity. One of them is the membrane-bound heterotrimeric guanine nucleotide-binding (G) protein-mediated signaling (Li et al., 2007). The Gα subunit of G protein upon activation regulates downstream effectors, such as adenylate cyclase, phospholipase, ion transporters, and mitogen-activated protein kinase (MAPK) involved in various biological processes including pathogenicity (Neves et al., 2002). Li et al. (2007) reported that two G proteins (Gβ and Gγ) regulate pathogenesis by monitoring the adenylate cyclase and MAP kinase pathway. *Rga1*, a Gα subunit gene, affects pathogenicity and its disruption decreased vegetative growth and pathogenicity of the rice sheath blight pathogen (Charoensopharat et al., 2008). The genome sequence of *R. solani* AG1-IA revealed that a group of secondary molecules including G protein-coupled receptors (GPCR), G protein subunits, MAPK pathway, cAMP pathway and calcium–calcineurin pathway genes may play a major role in pathogenesis (Zheng et al., 2013).

**TABLE 1:** List of effector molecules related to *R. solani* colonization in rice plant.

Effector Molecules	Properties	Function	Defense response compromised in rice plant	References
AGLIP1	Lipase	Signal peptide and active sites of AGLIP1 play a role in inducing cell death in rice protoplasts	flg22- and chitin-triggered PR genes expression suppressed	Li et al., 2019
RsPG2	Polygalacturonase (Cell-wall degrading enzyme)	release of reducing sugar and induce rice sheath tissue necrosis	Hydrolysis of the α-1, 4-glycosidic linkage of D-galacturonic acid in pectin in the plant cell-wall	Chen et al., 2017
AG1IA_04727	Polygalacturonase			Rao et al., 2019
α-1, 3-glucan	Polysaccharide	α-1, 3-glucan mask cell wall chitin of *R. solani* which is non-degradable in plants	Pattern Recognition Receptors in rice do not recognize α-1, 3-glucan masked chitin	Fujikawa et al., 2012
CAZYmes (Carbohydrate active enzymes)		cell wall degradation	Various glycoside hydrolases, glucosyl transferases, and polysaccharide lyases cause depolymerization of the host cell wall and colonization of the pathogen	Zheng et al., 2013; Ghosh et al., 2014
AG1IA_09161	Glycosyltransferase GT family 2 domain	Attachment of fungal pathogen and cell wall degradation		Zheng et al., 2013
AG1IA_05310	Cytochrome C oxidase assembly protein CtaG/cox11 domain	programmed cell death in host plant		

When a pathogen attacks a plant, the plant uses various pathways and defense mechanisms to prevent it from colonizing. On infection by *R. solani*, rice plants respond by activating various signaling pathways and producing antimicrobial compounds. The plant immune system is of two types, PTI (PAM- pathogen associated molecular triggered immunity) and ETI (effector-triggered immunity). PTI is the first line of defense in plants, which is initiated when pattern recognition receptors (PRRs) recognize non-self molecular patterns from pathogens. PTI induces a relatively weak immune response that restricts colonization by invading organisms. ETI, the second line of defense, is initiated when a cognate resistance (R) protein directly or indirectly recognizes highly variable pathogen molecules called avirulence (*Avr*) effectors and induces a hypersensitive reaction (Liu W. et al., 2014). Pathogenesis related proteins (PR proteins) are produced by the host plant only in pathological or related stress situations. PR3 and PR4 families of chitinases that hydrolyze the β-1,4 linkages between *N*-acetylglucosamine residues of chitin, a structural polysaccharide of the cell wall of *R. solani* are differentially induced in rice plants. Chitin fragments are recognized by LysM receptor-like proteins (Gust et al., 2012). POC1, a cationic pathogen-induced peroxidase is upregulated in rice on *R. solani* infection (Taheri and Tarighi, 2010). Most PRs are induced by the action of salicylic acid (SA), Jasmonic acid (JA), or ethylene (ET), and possess antimicrobial activities. A JA-deficient rice mutant, *Hebiba*, exhibited enhanced susceptibility to the sheath blight disease (Taheri and Tarighi, 2010). It was found that transgenic plants overexpressing *WRKY30* could improve disease resistance by accumulating more JA and conferred resistance to sheath blight by activating the JA/ET signaling cascade. Transcriptome analysis of sheath blight resistant and susceptible rice cultivars infected with *R. solani* led to the identification of 7624 differentially expressed genes (DEGs), mainly associated with cell wall, β-glucanase, respiratory burst, phenylpropanoids and lignin (Yuan et al., 2018; Molla et al., 2020).

## Management of Sheath Blight Disease

Currently, sheath blight disease of rice is largely managed through the use of fungicides, utilization of genetic resistance/tolerance, cultural practices and biological control are also strategically adopted in the integrated management. Although rice germplasm shows diverse responses to *R. solani* infection, yet, none of the rice varieties, landraces, weedy types or wild relatives have been identified as immune or completely resistant to this disease. However, some of the genotypes have been found to be partially resistant.

### Chemical Control

In the absence of effective host plant resistance against sheath blight pathogen in rice, the management of sheath blight disease is mainly carried out through the use of chemicals (Naik et al., 2017). Foliar spray and seed treatment are the most popular method of fungicidal application against *R. solani*. Even though both systemic and non-systemic fungicides are used for chemical management, systemic fungicides offer better management of this disease (Naik et al., 2017). Timely application of selective fungicides between panicle differentiation and heading stage offers effective protection against this disease. Periodical monitoring of the rice field and application of fungicides at the initial stages of infection especially at booting stage is recommended for managing sheath blight in susceptible varieties (Singh et al., 2016; Uppala and Zhou, 2018).

Several chemical formulations are in use for the control of sheath blight in rice (Table 2). The major focus in the development has been on the identification of fungicides with novel target sites and diverse modes of action. Presently, the Strobilurin group of systemic fungicides are the most preferred chemical group to manage sheath blight disease in rice (Yellareddygari et al., 2014). Strobilurin group of fungicides are derivatives of β-methoxy acrylates and are obtained from forest-grown wild mushrooms (*Strobilurus tenacellus*). Azoxystrobin from this group is very effective for not only controlling the disease but also found to enhance yield as well (Groth and Bond, 2007). Triazole fungicides are also commonly used in sheath blight management. Application of other chemicals such as Flutolanil, Carbendazim, Iprobenfos, Mancozeb, Thifluzamide and Validamycin also offers effective control of this disease.

**TABLE 2:** List of commercially used chemicals for managing sheath blight disease of rice.

Chemical group	Active ingredient (a.i.)	Trade name	Target site	Dosage* (g/ha)	References
Strobilurin	Azoxystrobin 23%EC	Amistar	Respiration: inhibition of Cytochrome bc1 at Quinone out site	125	Sanjay et al., 2012 Bag et al., 2016 FRAC, 2021
	Kresoxim-methyl	Sovran		250	
	Trifloxystrobin	Flint		150	
	Fluoxastrobin	Aftershock			
	Pyraclostrobin	insignia		75–100	
Triazole	Difenoconazole 25%EC	Score	Sterol biosynthesis in the cell membrane	62.5–125	Kandhari, 2007 Kumar et al., 2013 Naik et al., 2017 FRAC, 2021
	Hexaconazole 5% EC	Contaf		50	
	Flusilazole 40%EC	Cursor		120	
	Tebuconazole 25.9%EC	Folicure		187.5	
	Propiconazole 25%EC	Tilt		125	
Phenyl-benzamides	Flutolanil	Prostar	Respiration: an inhibitor of Succinate dehydrogenase	560	Kumar et al., 2013
Benzimidazoles	Carbendazim 50% WP	Bavistin	Cytoskeleton: assembling of ß-tubulin during mitosis	250	Prasad et al., 2006; Kandhari, 2007
Organophosphates	Iprobenfos 48%EC	Kitazin	Lipid synthesis: methyltransferase	240	Kumar et al., 2013
Dithiocarbamate	Mancozeb 35%SC	Dithane M-45	Multi-site contact activity	875	Prasad et al., 2006 FRAC, 2021
Carboxamide	Thifluzamide 24% SC	Spencer	Respiration: NADH oxidoreductase	375	Sunder et al., 2003
	Fluxapyroxad		Inhibition pathogen mycelial growth	100	Chen Y. et al., 2014
Phenylureas	Pencycuron 22.9%SC	Monceren	Cytoskeleton:—cell division	187.5	Kumar et al., 2013
Glucopyranosyl antibiotic	Validamycin	Sheathmar	Inhibition of trehalose	60	Miyagi, 1990
Nano Particle -Fungicides	Halogen substituted Azomethines		Tested effective against sheath blight		Siddhartha et al., 2020
	Silver and Gold Nanoparticle		Reduces the radial growth of pathogen		Das and Dutta, 2021

*Active ingredient (g/ha).

The use of a single chemical with the same mode of application for a prolonged time leads to the evolution of resistance in the fungus (Uppala and Zhou, 2018). Hence, a combinatory chemical formulation such as Azoxystrobin 18.2% + Difenoconazole 11.4% (Bhuvaneswari and Raju, 2012; Kumar et al., 2018); Propiconazole + Difenoconazole (Kandhari, 2007); Prothioconazole + Tebuconazole 240 g/kg SC (Chen et al., 2021). Captan 70% + Hexaconazole 5% (Pramesh et al., 2017); Trifloxystrobin 25% + Tebuconazole 50% (Shahid et al., 2014; Rashid et al., 2020); Carbendazim + Mancozeb (Prasad et al., 2006; Kumar et al., 2013); Carbendazim 25% + Flusilazole 12.5% SE (Sanjay et al., 2012) etc., are recommended to manage the disease. The chemical method of control is applicable for all areas, irrespective of varieties and has an advantage in a reduction in disease occurrence, spread and enhance yield. However, it has several disadvantages such as environmental hazards that could deteriorate soil health, and cause groundwater pollution. The toxic residue may enter the food chain affecting the health of both humans and animals. It is difficult for a new chemical to have a balancing role in disease management and environmental safety. Therefore, the use of non-chemical control options like cultural, biological, and development and use of resistant varieties offers a viable solution to sheath blight management.

### Cultural Practices

Historical records on varietal susceptibility, prior disease incidence, prevailing weather conditions and disease spread help in devising appropriate cultural practices for managing sheath blight disease of rice (Singh et al., 2019). Agro-morphological traits of rice including plant height, stem thickness and tiller angle, length and width of flag leaf, days to heading and planting density affect the susceptibility of rice to *R. solani*.

Plant height has been found to show a strong negative association between relative lesion length (Willocquet et al., 2012). Wider spacing reduces the sheath blight severity by improving the canopy thickness. Split application and use of slow-releasing nitrogenous fertilizers have been found to reduce sheath blight infection (Roy, 1986). The effect of dose of nitrogen fertilizer on disease spread has been higher than the effect of plant density (Zhang et al., 1995). Similar to nitrogen, higher doses of phosphorous fertilizers increase the disease incidence, while potassic fertilizers have been found to reduce it (Sarkar et al., 1991). Silicon application to rice fields through carbonized rice husk helps delay the disease spread without any negative effect on yield (Sabes et al., 2020). A waste product from charcoal production (Bamboo tar) was reported to inhibit multiple diseases including rice sheath blight (Maliang et al., 2021). Timely removal of weeds which are alternate host for *R. solani*, removal of plant debris, crop rotation with non-host crops reduces the sheath blight incidence by minimizing the primary inoculum sclerotia (Singh et al., 2019).

### Biological Control

In addition to chemical and cultural control, biological control has been suggested as a very promising strategy to manage necrotrophic fungus. Plant extracts or botanicals are very effective in managing the disease. Extracts from garlic, ginger, neem leaf and clove inhibit more than 80% mycelial growth in *R. solani* (Chakrapani et al., 2020; Rajeswari et al., 2020). Microbial antagonism is a common property found between microorganisms and it is most predominant among soil microbes. This effect of antagonism between the pathogen and beneficial microbes in the soil will lead to a reduction in disease development to a greater extent. There are several biocontrol agents (BCAs) belonging to actinomycetes, fungi and bacteria. Actinomycetes colonize the plant roots and represent a greater portion of the rhizosphere microflora. Actinomycetes against *R. solani* in tomatoes could reduce the disease incidence by up to 63% (Singh et al., 2017). One of the most common actinomycetes, *Streptomyces* spp. is reported to reduce the growth of *R. solani* up to 50% and disease suppression up to 53.3% (Patil et al., 2010). Ethyl acetate extracted from *Streptomyces diastatochromogenes*, KX852460 have been found to inhibit mycelial growth, reduce sclerotia formation and suppress lesion length on *R. solani* AG3 (Ahsan et al., 2019). Another group of potential BCAs mostly used against *Rhizoctonia* is fungal antagonists. Many species of *Trichoderma, Corticium, Aspergillus* and *Gliocladium* have been used for managing sheath blight disease (Chinnaswami et al., 2021). For effective management, these BCAs are applied as a soil treatment, foliar spray and root dipping of seedlings. Different strains of *Trichoderma* have been reported to inhibit *Rhizoctonia* growth by up to 71% and reduce the sheath blight infestation by up to 59% (Mishra et al., 2020). *Trichoderma* can be applied alone or in combination with other BCAs like Vesicular arbuscular mycorrhiza, *Pseudomonas* and yeasts for both controlling the pathogen and supplementing growth factors (Mathivanan et al., 2005; Mohammed et al., 2020). Plant growth-promoting rhizobacteria (PGPR) are the most common group of bacterial BCAs used against a wide range of plant pathogens for disease reduction. PGPR also helps in increasing root growth, phosphate solubilization, nitrogen uptake, iron-chelating siderophores and phytohormone synthesis. Among the different PGPR, *Pseudomonas* and *Bacillus* provide an effective way of systemic resistance against sheath blight. Rice seedlings treated with different strains of *Pseudomonas fluorescence* helped to increase the chitinase activity responsible for the suppression of sheath blight disease (Radjacommare et al., 2004). *Bacillus sp.* having a broad range of antibiotic properties was also very useful in reducing the growth of *Rhizoctonia* (Abbas et al., 2019; Raj et al., 2019). The combination of *Bacillus subtilis* strain MBI600 with Azoxystrobin helps not only disease suppression but also increases the yield to 14% (Zhou et al., 2021). In a recent study, three strains of nitrogen-fixing cyanobacteria have been reported to significantly inhibit the growth of *R. solani* (Zhou et al., 2020). However, the effectiveness of BCAs in sheath blight is influenced by their ability to survive, multiply and control pathogens and also provide additional supplements promoting rice growth. Nanoparticles of Gold and Silver have antifungal activity against *R. solani* (Das and Dutta, 2021). Recently, silver nanoparticles from rice leaf extract have been reported to be very effective against *R. solani* infection in rice (Kora et al., 2020). Different biocontrol agents were screened against sheath blight for their timing of application in a greenhouse environment, treatment of these bio fungicides before pathogen inoculation has a great role against the disease (Tuyen and Hoa, 2022). Eugenol from clove (*Syzygium aromaticum L*.) has been found to control this pathogen by dehydrating the cell and increasing the cell membrane permeability (Zhao et al., 2021).

## Crop Improvement Strategies Against *R. Solani*

Theoretically breeding for sheath blight resistance is mainly based on two approaches, disease escape and disease resistance. Disease escape mainly consists of plant architectural traits including plant height, heading date and stem thickness (Sattari et al., 2014; Susmita et al., 2019). The standard protocol for screening for disease resistance is based on relative lesion height (RLH) which is calculated in the percentage of ratio lesion height to plant height (Sharma et al., 1990; IRRI, 1996). Conventional breeding is more difficult in this case because of the direct influence of plant height on RLH during its screening protocol. Hence marker assisted breeding is highly preferred for the introgression of identified resistance QTLs. Marker assisted breeding has several advantages over conventional breeding as it helps in accurate selection of desired genotypes, saves time during selection, reduces linkage drag during introgression of genomic regions and helps in easier gene pyramiding.

### Donors for Resistance

Development of resistant rice varieties through genetic improvement is a sustainable option for managing plant diseases. Since there are no genotypes with absolute resistance, identification of reliable resistance sources must be confined to the moderate to high levels of tolerance in the germplasm. There are several such genotypes reported (Table 3) that are being used in breeding sheath blight resistant cultivars. Among the cultivated species, the *indica* cultivars are reported to show better resistance than the *japonica* type (Liu et al., 2009; Willocquet et al., 2012). Additionally, some accessions of wild species such as *O. rufipogon, O. nivara, O. meridionalis* and *O. barthii* have been reported to be resistant to sheath blight disease (Prasad and Eizenga, 2008; Bashyal et al., 2017).

**TABLE 3:** Rice genotypes identified as sources of resistance to sheath blight disease.

Source of resistance	References
Dudsor, NC 678, Bhasamanik	Das, 1970
Zenith, Chin-Kou-tsan, CO17	Wu, 1971
Lalsatkara	Roy, 1977
ARC 18119, ARC15762	Bhaktvatsalam et al., 1978
Jaya, IR24, IR26, IR29, Mashoori, Jagganath	Rajan and Nair, 1979
Tapachoor, Laka, Bahagia	Crill et al., 1982
Tapoo cho Z, Tetep, Bharati Rohini	Gokulapulan and Nair, 1983
Chidon, Dholamula, Supkheru, Taraboli 1	Borthakur and Addy, 1988
Tetep	Sha and Zhu, 1989 Channamallikarjuna et al., 2010
BPT-6, BogII, MTU 3, MTU 3642, MTU7, MTU 13, Saket, Arkavati, Aduthurai	Ansari et al., 1989
LSBR 33, LSBR 5	Xie et al., 1992
TIL 642, TIL 455, TIL 514	Singh and Dodan, 1995
Teqing	Li et al., 1995; Pinson et al., 2005
Mairan KK2, As 93-1, Camor, Dodan, IR40, Chingdar	Marchetti et al., 1996
Jasmine 85	Pan et al., 1999; Zou et al., 2000; Li et al., 2009
Mairan, Panjasali, N-22, Chingdar, Upland 2, AS93-1	Singh and Borah, 2000
Minghui 63	Han et al., 2002
Zhaiequing 8, Jingxi 17	Kunihiro et al., 2002
Xiangzaoxian 19	Che et al., 2003
WSS2	Sato et al., 2004
*O. latifolia*; DRW 37004, WR 106, DRW 21009, DRW 24008	Ram et al., 2008
*O. nivara*; IRGC 104443, IRGC 104705, IRGC 100898 *O. officinalis*; IRGC 105979 *O. meridionalis*; IRGC 105306 *O. barthii*; IRGC 100223	Prasad and Eizenga, 2008
C418	Chen et al., 2009
Pecos	Sharma et al., 2009
YSBR1	Zuo et al., 2009
Baiyeqiu	Xu et al., 2011
RSB03	Fu et al., 2011
GSOR 310389, GSOR 31147, GSOR 310475	Jia L. et al., 2012
LJRIL103, LJRIL158, LJRIL186, LJRIL220	Jia Y. et al., 2012
MCR10277	Nelson et al., 2012
Jarjan, Nepal 8, Nepal 555	Taguchi-Shiobara et al., 2013
HJX74	Zhu et al., 2014
Kajrahwa, BML 21-1, BPL 7-12, BML 27-1	Dubey et al., 2014
RSB02	Liu Y. et al., 2014
*O. meridionalis*; IRGC105608	Eizenga et al., 2015
ARC10531	Yadav et al., 2015
2F18-7-32 (32R)	Gaihre et al., 2015
Yangdao 4	Wen et al., 2015; Yuan et al., 2019
TN1	Zeng et al., 2015
Phougak, Gumdhan, Ngnololasha, Wazuhophek, SM 801, 10-3	Dey et al., 2016
*O. rufipogon;* IC336719, IC336721	Bashyal et al., 2017
Dagad Deshi	Koshariya et al., 2018; Mandal et al., 2018
Bico Branco, DOM Zard, Vary Vato462, T26, Peh-Kuh- Tsao, Bombilla, Koshihikari, PR304, Kaukau, Ghati Kmma Nangarhar	Chen Z. et al., 2019

### Genetics and Analysis of Quantitative Resistance

**FIGURE 4:**
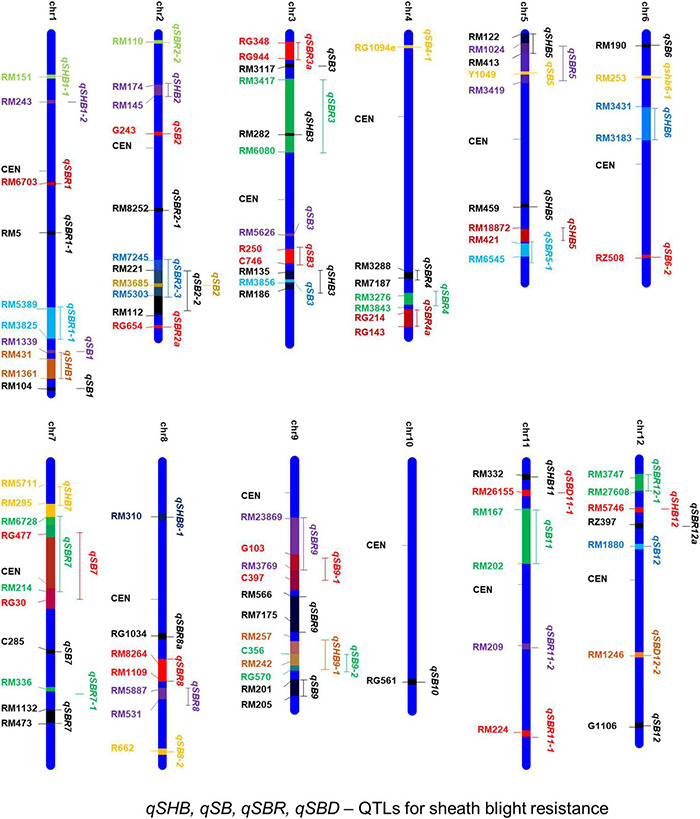
A consolidated chromosomal map showing the QTLs mapped and the markers linked to sheath blight resistance in rice. *qSHB, qSB, qSBR*, and *qSBD* indicate the quantitative trait loci for sheath blight disease resistance.

Several earlier studies indicate that the tolerance against sheath blight disease in rice is a quantitative trait governed by polygenes (Xu et al., 2011; Koshariya et al., 2018). Therefore, it is essential to map the genomic regions governing quantitative variation for tolerance among the source germplasm. Attempts on mapping quantitative trait loci (QTLs) have been taken up in rice for sheath blight tolerance. One of the earliest attempts by Li et al. (1995) used RFLP markers in an F_4_ population derived from Lemont/Teqing. Lemont was a highly susceptible *japonica* cultivar, while Teqing was a semidwarf high yielding Chinese *indica* variety with high tolerance to leaf blight. Since then a large number of QTLs governing resistance to sheath blight disease have been reported across all the 12 chromosomes of the rice genome (Table 4). A map showing the physical location of the reported QTLs and the linked markers is presented in Figure 4. Most of the earlier mapping populations were based on the partially resistant *indica* genotypes such as Teqing and Jasmine 85 and the susceptible *japonica* genotype, Lemont (Li et al., 1995; Pan et al., 1999; Wen et al., 2015). Using these mapping populations, a large number of QTLs governing sheath blight resistance have been mapped (Li et al., 1995; Zou et al., 2000; Liu et al., 2009; Eizenga et al., 2015). Eizenga et al. (2013, 2015) also have identified resistance sources from wild accessions of *O. nivara* and *O. meridionalis*. QTLs for resistance have been mapped from weedy rice also (Goad et al., 2020; Jia et al., 2022). Goad et al. (2020) reported four QTLs from RIL populations generated by crossing the rice cultivar, Dee-Geo-Woo-Gen (DGWG) with two weed species (straw hull and black hull awned). Yuan et al. (2019) utilized a RIL population from Lemont/Yangdao4 to map 128 minor effect QTLs, most of which clustered around 17 stable loci across the rice genome.

**TABLE 4:** List of QTLs mapped for sheath blight resistance in rice.

QTLs	Chr.	Linked markers	Mapping Population	Cross	References
*qSB1* *qSB1* *qSB1* *qSBR1-1* *qSHB1* *qSBR1-1* *qSHB1* *qSB1-1* *qSBR1* *qSHB1-1* *qSHB1-1* *qSHB1-2* *qSBR1-1* *qSBR1-2* *qSHB1-2*	1	RG532X RM104 RM1339 HvSSR68 RM431-RM12017 RM5389-RM3825 RM1361- RM104 InDel Markers RM6703-RM5448 RM151-RM12253 HvSSR1-87 RM243 RM5 RM84 SNP	RIL RIL F_2:3_ RIL DH RIL BC_2_F_1_ CSSL F_2_ F_2_ and BC_1_F_2_ RIL RIL RIL RIL RIL	Lemont/Teqing Lemont/Jasmine 85 Rosemont/Pecos HP2216/Tetep Maybelle/Baiyeqiu HH1B/RSB03 IRGC100898/Bengal HJX74/Amol3 32R/Nipponbare BPT-5204/ARC 1053 Danteshwari/Dagad Deshi Danteshwari/Dagad Deshi Danteshwari/Dagad Deshi Danteshwari/Dagad Deshi SHW and BHAW/Dee-Geo-Woo-Gen	Pinson et al., 2005 Liu et al., 2009 Sharma et al., 2009 Channamallikarjuna et al., 2010 Xu et al., 2011 Fu et al., 2011 Eizenga et al., 2013 Zhu et al., 2014 Gaihre et al., 2015 Yadav et al., 2015 Koshariya et al., 2018 Koshariya et al., 2018 Mandal et al., 2018 Mandal et al., 2018 Goad et al., 2020
*qSBR2 a* *qSB2* *qSBR2* *qSB2* *qSHB2* *qSBR2-1* *qSBR2-2* *qSBR2-3* *qSBR2-1* *qSBR2-2* *qSB2-2*	2	RG654-RZ260 G243-RM29 RM3685 RM174-RM145 RM5340-RM521 RM110-Osr14 RM7245-RM5303 RM8254-RM8252 RM3857-RM5404 RM221-RM112	F_4_ F_2_ DH F_2:3_ DH RIL RIL RIL DH DH RIL	Lemont/Teqing Jasmine 85/Lemont Zhai Ye Qing 8/Jing Xi 1 Rosemont/Pecos Maybelle/Baiyeqiu HH1B/RSB03 HH1B/RSB03 HH1B/RSB03 MCR10277/Cocodrie MCR10277/Cocodrie Lemont/Jasmine 85	Li et al., 1995 Pan et al., 1999 Kunihiro et al., 2002 Sharma et al., 2009 Xu et al., 2011 Fu et al., 2011 Fu et al., 2011 Fu et al., 2011 Nelson et al., 2012 Nelson et al., 2012 Liu et al., 2013
*qSBR3a* *qSB3* *qSBR3q*	3	RG348-RG944 R250-C746	F_4_ F_2_ DH	Lemont/Teqing Jasmine 85/Lemont Zhai Ye Qing 8/Jing Xi 1	Li et al., 1995 Pan et al., 1999 Kunihiro et al., 2002
*qSB3* *qSB3* *qSB3* *qSBR3-1* *qSHB3* *qSHB3* *qSBR3* *qSBD3-1* *qSHB3*	3	RM3856 RM5626 RM3117 RM251 RM135-RM186 RM232-RM282 RM3417-RM6080 D328B-D331B RM232	BC_1_F_1_ RIL F_2:3_ RIL DH BC_2_F_1_ F_2_ F_2_ and F_2:3_ RIL	Hinohikari/WSS2//hinohikari Lemont/Jasmine 85 Rosemont/Pecos HP2216/Tetep Maybelle/Baiyeqiu IRGC100898/Bengal 32R/Nipponbare Yangdao 4/Lemont Danteshwari/Dagad Deshi	Sato et al., 2004 Liu et al., 2009 Sharma et al., 2009 Channamallikarjuna et al., 2010 Xu et al., 2011 Eizenga et al., 2013 Gaihre et al., 2015 Wen et al., 2015 Koshariya et al., 2018
*qSBR4a* *qSB4-1* *qSBR4* *qSBR4* *qSBR4-1* *q SHB4*	4	RG143-RG214 RG1094e RM3288-RM7187 RM3276-RM3843 RM273 SNP	F_4_ RIL RIL F_2_ RIL RIL	Lemont/Teqing Lemont/Teqing HH1B/RSB03 32R/Nipponbare Danteshwari/Dagad desi SHW and BHAW/DGWG//DGWG	Li et al., 1995 Pinson et al., 2005 Fu et al., 2011 Gaihre et al., 2015 Mandal et al., 2018 Goad et al., 2020
*qRsb 1* *qSB5* *qSB5* *qSBR5-1* *qSHB5* *qSHB5* *qSBR5* *qSBR5-1* *qSHB5*	5	RM 39300 Y1049 RM13 RM421-RM6545 RM18872-RM421 RM122-RM413 RM1024-RM3419 HvSSR5-52 RM459	F_2_ RIL RIL RIL DH BC_2_F_1_ F_2_ RIL RIL	4011/Xiangzaoxian19 Lemont/Teqing Lemont/Jasmine 85 HH1B/RSB03 Maybelle/Baiyeqiu IRGC100898/Bengal 32R/Nipponbare Danteshwari/Dagad desi Danteshwari/Dagad Deshi	Che et al., 2003 Pinson et al., 2005 Liu et al., 2009 Fu et al., 2011 Xu et al., 2011 Eizenga et al., 2013 Gaihre et al., 2015 Mandal et al., 2018 Koshariya et al., 2018
*qSB6-2* *qSB6* *qSBR6-1* *qShB6* *qSHB6-1*	6	RZ508 RM190 HvSSR6-35 RM3183–RM541 RM400-RM253	RIL RIL RIL BC_2_F_1_ F_2_ and BC_1_F_2_	Lemont/Teqing Lemont/Jasmine 85 Danteshwari/Dagad desi IRGC100898/Bengal BPT 5204/ARC 1053	Pinson et al., 2005 Liu et al., 2009 Mandal et al., 2018 Eizenga et al., 2013 Yadav et al., 2015
*qSB7* *qSBR7* *qSB7*	7	RG30-RG477 C285 RM336	F_2_ DH RIL	Jasmine 85/Lemont Zhai Ye Qing 8/Jing Xi 1 Lemont/Teqing	Pan et al., 1999 Kunihiro et al., 2002 Pinson et al., 2005
*qSBR7-1* *qSBR7* *qSHB7* *qSBR7* *qSHB7-1* *qSHB7-2* *qSHB7-3* *qSBL7*		RM1132-RM473 RM295-RM5711 RM6728-RM214 RM81-RM6152 RM10-RM21693 RM336-RM427 D760-RM248	RIL RIL BC_2_F_1_ F_2_ F_2_ and BC_1_F_2_ F_2_ and BC_1_F_2_ F_2_ and BC_1_F_2_ F_2_ and F_2:3_	HP2216/Tetep HH1B/RSB03 IRGC100898/Bengal 32R/Nipponbare BPT-5204/ARC 1053 BPT-5204/ARC 1053 BPT-5204/ARC 1053 Yangdao 4/Lemont	Channamallikarjuna et al., 2010 Fu et al., 2011 Eizenga et al., 2013 Gaihre et al., 2015 Yadav et al., 2015 Yadav et al., 2015 Yadav et al., 2015 Wen et al., 2015
*qSBR8a* *qSB8-2* *qSBR8-1* *qSBR8* *qSBR8* *qSHB8-1*	8	RG20-RG1034 R662 RM210 RM8264-RM1109 RM5887-RM531 RM21792-RM310	F_4_ RIL RIL RIL F_2_ F_2_ and BC_1_F_2_	Lemont/Teqing Lemont/Teqing HP2216/Tetep HH1B/RSB03 32R/Nipponbare BPT-5204/ARC 1053	Li et al., 1995 Pinson et al., 2005 Channamallikarjuna et al., 2010 Fu et al., 2011 Gaihre et al., 2015 Yadav et al., 2015
*qSBR9a* *qSB9-1* *qSB9-2* *qSB9* *qSB9-2* *qSB9* *qSBR9-1* *qSBR9* *qSBR9-1* *qSBR9* *qSHB9-2* *qSBR9*	9	RG9 10b-RZ777 C397-G103 RG570-C356 RM205-RM201 RM245 RM3823 RM257 RM23869-RM3769 RM24708-RM3823 Nag08KK18184-Nag08KK18871 RM 257-RM107 RM566-RM7175	F_4_ F_2_ F_2_ F_2_ RIL F_2:3_ RIL RIL DH BIL BC F_2_	Lemont/Teqing Jasmine 85/Lemont Jasmine 85/Lemont Teqing/Lemont Lemont/Jasmine 85 Rosemont/Pecos HP2216/Tetep HH1B/RSB03 MCR10277/Cocodrie Jarjan/Koshihikari//Koshihikari IRGC105608/Lemont 32R/Nipponbare	Li et al., 1995 Zou et al., 2000 Zou et al., 2000 Tan et al., 2005 Liu et al., 2009 Sharma et al., 2009 Channamallikarjuna et al., 2010 Fu et al., 2011 Nelson et al., 2012 Taguchi-Shiobara et al., 2013 Eizenga et al., 2015 Gaihre et al., 2015
*qSHB9-1* *qSHB9-2* *qSHB9-3* *qSBR9-1*	9	RM257-RM242 RM205-RM105 RM24260-RM 3744 RM444	F_2_ and BC_1_F_2_ F_2_ and BC_1_F_2_ F_2_ and BC_1_F_2_ RIL	BPT-5204/ARC 1053 BPT-5204/ARC 1053 BPT-5204/ARC 1053 Danteshwari/Dagad desi	Yadav et al., 2015 Yadav et al., 2015 Yadav et al., 2015 Mandal et al., 2018
*qSB10*	10	RG561	RILK	Lemont/Teqing	Pinson et al., 2005
*qSB11* *qSB11* *qSBR11-1* *qSBR11-2* *qSBR11-3* *qSHB11* *qSB11* *qSBD11-1*	11	G44-RG118 RM167-Y529 RM224 RM209 RM202 RM332-RM21 InDel Markers D1103-RM26155	F_2_ F_2_ RIL RIL RIL BC_2_F_1_ CSSL F_2_ and F_2:3_	Jasmine 85/Lemont Teqing/Lemont HP2216/Tetep HP2216/Tetep HP2216/Tetep IRGC100898/Bengal HJX74/Amol3 Yangdao 4/Lemont	Zou et al., 2000 Tan et al., 2005 Channamallikarjuna et al., 2010 Channamallikarjuna et al., 2010 Channamallikarjuna et al., 2010 Eizenga et al., 2013 Zhu et al., 2014 Wen et al., 2015
*qSBR12a* *qSB12* *qSB12* *qSBR12-1* *qSHB12* *qSBD12-2* *qSHB12-1* *qSHB12-2* *qSBR12-1*	12	RG214a-RZ397 RM1880 G1106 RM3747-RM27608 RM5746-RM277 RM1246-D1252 RM260 RM277 RM20	F_4_ BC_1_F_1_ RIL DH BC_2_F_1_ F_2_ and F_2:3_ RIL RIL RIL	Lemont/Teqing Hinohikari/WSS2//hinohikari Lemont/Teqing MCR10277/Cocodrie IRGC100898/Bengal Yangdao 4/Lemont Danteshwari/Dagad Deshi Danteshwari/Dagad desi Danteshwari/Dagad desi	Li et al., 1995 Sato et al., 2004 Pinson et al., 2005 Nelson et al., 2012 Eizenga et al., 2013 Wen et al., 2015 Koshariya et al., 2018 Mandal et al., 2018 Mandal et al., 2018

*SHW, Straw hull weed; BHAW, Black hull awned weed; DGWG, Dee Geo Woo Gen.*

### Genome Wide Association Studies

Identification of genomic regions associated with sheath blight resistance has also been carried out using genome wide association studies but on a limited scale. Jia L. et al. (2012) identified 10 marker-trait associations (MTAs) and three genotypes for resistance from a set of 217 core entries from USDA using 155 genome-wide simple sequence repeat (SSR) markers. Using a larger population of 456 rice accessions, Sun et al. (2014) identified 10 significant MTAs with 144 SSR markers. Chen Z. et al. (2019) reported 11 MTAs and two QTLs, *qSB3* and *qSB6* by screening 299 rice varieties with 44K SNPs. GWAS with 228 rice accessions genotyped with 700,000 SNPs identified two major MTAs associated with sheath blight resistance in rice (Oreiro et al., 2020). Zhang et al. (2019) identified 562 MTAs for lesion height, 134 for culm length and 75 MTAs for relative lesion height through GWAS on a set of 563 rice accessions genotyped with 220,335 SNPs. GWAS was conducted using 259 diverse verities and identified a regulation model against the disease (Wang et al., 2021).

### Fine Mapping of QTLs

Although a large number of major and minor effect QTLs have been identified for sheath blight resistance in rice, efforts to fine map these QTLs have been limited. Chromosome segment substitution lines (CSSLs) are a group of homozygous lines, each having a different chromosome segment from the donor species. Individually one CSSL has a donor segment that overlaps the other donor segment in the next CSSL. Altogether, CSSLs contain the whole genomic DNA of donor species in different segment-wise. The CSSLs eliminate the genetic background effect, and enables, the fine mapping of QTLs (Eshed and Zamir, 1994). Channamallikarjuna et al. (2010) fine mapped a major QTL, *qSBR11-1* for sheath blight resistance, which has been narrowed down to 0.85 Mb on chromosome 11. A set of 154 putative genes have been identified within this genomic region, out of which 11 chitinase genes in tandem repeats have been identified as candidate genes governing resistance to sheath blight disease. A major QTL *qSB-11*^LE^** identified from the first QTL mapping effort (Li et al., 1995) and subsequent studies (Zou et al., 2000; Tan et al., 2005) has been fine mapped to a 78.8 kb genomic region, from which three candidate genes have been identified (Zuo et al., 2013). *qSB-9*^TQ^** from Teqing has also been fine mapped to a region of 146 kb region using CSSLs (Zuo et al., 2014b).

### Marker Assisted Breeding for Sheath Blight Resistance in Rice

Mapping and validation of QTLs are essential for their utilization in marker assisted breeding. Teqing is one of the most frequent donors for the QTLs, *qSB7*^TQ^*, qSB9*^TQ^** and *qSB12^TQ^*. Marker assisted introgression of single or multiple of these QTLs were found to reduce the yield loss due to sheath blight disease (Wang et al., 2012; Chen Z. X. et al., 2014). Sheath blight resistance has been enhanced by the introgression of QTL, *qSB9^TQ^* along with QTL for tiller angle, *TAC1^TQ^* (Zuo et al., 2014a). Yin et al. (2008) introgressed three main effect QTLs namely, *qSB7*^TQ^*, qSB9*^TQ^** and q*SB11^LE^* into Lemont to develop sheath blight resistant genotypes. In India, Tetep has been widely used as a donor source for both sheath blight as well as blast resistance. A major QTL, *qSBR11-1* using ‘Tetep’ was introgressed along with another gene, *Pi54* governing blast resistance into a bacterial blight resistant Basmati rice variety, ‘Improved Pusa Basmati 1’ leading to the development of improved near isogenic lines (NILs) with resistance to virulent strains of *R. solani* (Singh et al., 2012). *qSBR11-1* and *Pi54* have been pyramided into the high yielding variety, CO51 (Senthilvel et al., 2021). Gene(s) for multiple diseases resistance including bacterial leaf blight *(xa5* + *xa13* + *Xa21*), Blast (*Pi54*) and sheath blight (*qSBR7-1* + *qSBR11-1* + *qSBR11-2*) have been pyramided into the background of popular cultivar ASD 16 and ADT 43 using, Tetep and IRBB60 as donors (Ramalingam et al., 2020). Raveendra et al. (2020) introgressed sheath blight resistance from Tetep into the background of bacterial blight resistant genotypes, CB14004 and CB14002.

### Biotechnological Approaches for Managing Sheath Blight Diseases of Rice

Comparison of transcripts between resistant and susceptible cultivars in response to *Rhizoctonia* led to the identification of Ethylene-insensitive protein 2, *trans*-cinnamate-4- monooxygenase and WRKY 33 transcriptome factor (Shi et al., 2020). Rice is endowed with resources and techniques enabling the study of the expression of these pathogen-related (PR) genes, anti-fungal genes and master genes for defense response affecting *R. solani* growth.

**FIGURE 5:**
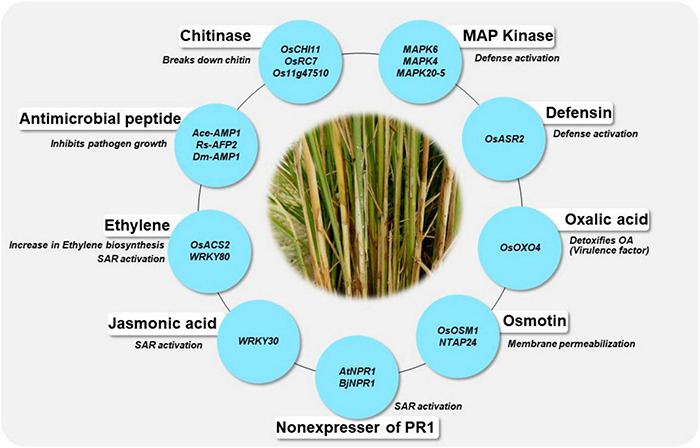
Genes are being utilized for the development of transgenics and their mode of action in conferring resistance to sheath blight disease of rice. The blue circle indicates the genes, the details of their mode of action are given in Table 5.

In the absence of stable sources of sheath blight resistance, genetic engineering offers promise in developing novel resistance in rice. Several potential genes from various species have been identified as candidates for engineering resistance against *Rhizoctonia solani* in rice (Table 5). Chitinase and glucanase are the most widely used genes for engineering resistance against *R. solani*. Lin et al. (1995) were the first to generate a transgenic line with constitutive expression of a chitinase gene (Chi11) leading to resistance to sheath blight disease of rice. Since then, many studies have demonstrated the effect of overexpression of the chitinase gene in rice. Chitinase cleaves at the β-1,4-glycosidic linkage of *N*-acetyl-D-glucosamine and glucanase cleaves at the β-1,3 linkage of glucan polymer, arresting the fungal invasion of the host tissues. Recent studies on overexpression of genes like *WRKY13* (Lilly and Subramanian, 2019), *OsBR2* (Maeda et al., 2019), *RGB1* and *RGG1* (Swain et al., 2019), *LPA1* (Sun et al., 2019a,^2020^) and *OsGSTU5* (Tiwari et al., 2020) have demonstrated the effectiveness of these genes in managing sheath blight of rice. Overexpression of the genes from the *WRKY* gene family namely, *OsWRKY4* (Wang et al., 2015a), *OsWRKY13* (Lilly and Subramanian, 2019), *OsWRKY30* (Peng et al., 2012) and *OsWRKY80* (Peng et al., 2016) have been reported to reduce *R. solani* infection in rice. A schematic representation of genes being utilized in the development of transgenics with resistance to sheath blight disease along with their mode of action is presented in Figure 5. Constitutive expression of *Chi11* and β*-1,3 glucanase* genes in a transgenic line, Pusa Basmati-CG27, helped to validate their role in conditioning sheath blight resistance, based on which these genes were used in marker assisted improvement of White Ponni (Kannan et al., 2017). Over expression of a basic helix–loop–helix transcription factor (*OsbHLH057*) with *cis*-acting *AATCA* has been reported to be effective against both sheath blight and drought (Liu et al., 2022). Recently, Dauda et al. (2022) identified a set of Cytokinin glucosyltransferases (CGTs) in rice with the plant secondary product glycosyltransferases (PSPG) motif of 44-amino-acid consensus sequence characteristic of plant uridine diphosphate (UDP)-glycosyltransferases (UGTs), the validation of which showed upregulation of four genes namely *LOC_Os07g30610.1, LOC_Os04g25440, LOC_Os04g25490*, and *LOC_Os04g25800* specifically under *R. solani* infection.

**TABLE 5:** Genes reported for sheath blight resistance in rice.

Group	Gene name	Function	References
Chitinase	*OsCHI11*	Degrades chitin by breaking β-1, 4 linkages	Lin et al., 1995 Sridevi et al., 2003
	*OsRC7*		Datta et al., 2001
	*RCH10*		Kim et al., 2003
	*Os11g47510*		Richa et al., 2017
Antimicrobial peptide	*pin A, pin B*	Plant defensin that inhibits pathogen growth	Krishnamurthy et al., 2001
	*Ace-AMP1*		Patkar and Chattoo, 2006
	*Dm-AMP1, Rs -AFP2*		Jha and Chattoo, 2009
	*RS-AFP2*		Jha and Chattoo, 2010
	*snakin-1*		Das et al., 2021
WRKY transcription factor	*OsWRKY30*	Positively regulated defense response	Peng et al., 2012
	*OsWRKY4*		Wang et al., 2015a
	*OsWRKY80*		Peng et al., 2016
	*OsWRKY13*		Lilly and Subramanian, 2019
	*OsWRKY53*	Negatively regulated	De Yuan et al., 2020
	*OsWRKY45*		Shimono et al., 2012
Osmotin	*ap24*	Plant defense response and Permeability stress	Rao et al., 2011
	*OsOSM1*		Xue et al., 2016
Oxalate oxidase	*Osoxo4*	Degrade oxalic acid (OA) and reduce the OA accumulation	Karmakar et al., 2016
	*OxDC*		Qi et al., 2017
Polygalacturonase (PG) inhibiting proteins (PGIP)	*OsPGIP1*	Stabilizes the plant cell wall component Pectin	Wang et al., 2015b
	*OsPGIP2^L233F^*		Chen X. J. et al., 2019
	*ZmPGIP3*		Zhu et al., 2019
Mitogen-activated protein (MAP) Kinases	*OsMAPK20-5*	Plant defense response	Liu et al., 2019
Thaumatin-like protein	*Tlp-D34*	Co-expression of *Tlp* with *Chi reduces disease index*	Shah et al., 2013
Ethylene biosynthetic genes	*OsACS2*	Overexpression of ethylene leads to resistance	Helliwell et al., 2013
Non-expressor of pathogenesis related gene	*AtNPR1* *BjNPR1*	Regulator of Systemic Acquired Resistance	Sadumpati et al., 2013 Molla et al., 2016
Sugar transporter	*OsSWEET11*	Negatively regulated	Gao et al., 2018
	*OsSWEET2a*		Gao et al., 2021
	*OsSWEET14*	Positively regulated	Kim et al., 2021
Loose Plant Architecture (LPA)	*LPA1*	Over expression	Sun et al., 2020 Chu et al., 2021
	*DEP1*	Dense and erect panicle	Liu et al., 2021
Defense associated protein	*OsGSTU5*	Over expression of tau class glutathione-S-transferase	Tiwari et al., 2020
Acyl-CoA-binding protein	*OsACBP5*	Overexpression of *OsACBP5* leads to resistance	Panthapulakkal et al., 2020
Kinesin like protein	*KSP*	*KSP* overexpression is less susceptible to disease	Chu et al., 2021
DNA-binding one finger (DOF) Transcription factor	*DOF11*	Activation of DOF leads to resistance	Kim et al., 2021
Probenazole responsive protein	*OsRSR1*	Enhanced disease resistance *via* NBS-LRR	Wang et al., 2021
Protein Phosphatase	*PP2A-1*	Overexpression leads to resistance	Lin et al., 2021
Non-host resistance gene	*IMPA 2*	Importin alpha (IMPA) 2 provides immunity	Parween and Sahu, 2022
Chlorophyll degradation gene	*OsNYC3*	Gene suppression leads to resistance	Cao et al., 2022

Small RNAs (siRNAs and miRNAs) play a major role in regulating several processes in plants by switching genes on and off leading to resistance to biotic/abiotic stresses. Host-induced gene silencing or RNA interference (RNAi) strategy has been utilized against *Rhizoctonia* by targeting pathogenicity linked MAP kinase genes (Tiwari et al., 2017) and polygalacturonase genes (Rao et al., 2019). Overexpression of a siRNA (SiR109944) targeting a gene, F-Box domain and LRR-containing protein 55, has been found to increase the susceptibility of rice to sheath blight disease (Qiao et al., 2020). An ethylene signaling gene, *EIL1* has been found to positively regulate sheath blight resistance in rice (Sun et al., 2019b). Transcriptome analysis has revealed that the upregulation of genes controlling cytoskeleton, membrane integrity, and glycolytic pathway plays a major role in disease resistance (Samal et al., 2022). It is recently reported that lauric acid has a role against *R. solani* by modifying fatty acid metabolism leading to apoptosis (Wang et al., 2022).

## Conclusion

Sheath blight is one of the diseases of major concern in rice with the potential to upset rice production and productivity. The causal agent, *R. solani* is a dynamic pathogen with a wide host range which enables it to overwinter and survive. *R. solani* has many anastomosis groups, among which AG1-IA is important as the rice sheath blight pathogen. Because of its versatility, the pathogen is very difficult to manage. Chemical control has been the most commonly used approach for management, which is not only environmentally unsafe but also leads to the evolution of novel virulent strains of the pathogen. Although there are other approaches such as cultural practices, and biological control to reduce the disease severity, utilizing host plant resistance is the most sustainable approach for managing this fungal disease. However, rice lacks absolute resistance to rice sheath blight, therefore moderate to high level of tolerance should be tapped as the source of resistance. There have been efforts to map QTLs among the tolerant lines, and many of them have been utilized in marker assisted breeding. However, the progress in molecular breeding has been slow as compared to other major diseases such as bacterial blight and blast diseases where effective genes have been widely available. Standard method of screening for sheath blight disease is based on relative lesion height (RLH) as given by IRRI. This RLH is directly influenced by plant height. Therefore, there is a need to develop a new method for screening against the disease with appropriate standardization. The breeding for sheath blight resistance also needs to focus on utilizing the QTLs through marker assisted introgression into popular cultivars. Several genes have been identified and some of them have been functionally characterized in rice and from other plant species, which provides an opportunity for the development of transgenics as well as genome-editing to create novel variations for managing the sheath blight of rice.

## Data Availability Statement

The original contributions presented in the study are included in the article/supplementary material, further inquiries can be directed to the corresponding author/s.

## Author Contributions

SK, AS, and KV proposed the idea. BB, MS, HB, and PB outlined the review. MS, AT, NS, and PC collected the materials and prepared the draft. RE, BB, SK, and KV edited the manuscript. All authors read and confirmed the final manuscript.

## Conflict of Interest

The authors declare that the research was conducted in the absence of any commercial or financial relationships that could be construed as a potential conflict of interest.

## Publisher’s Note

All claims expressed in this article are solely those of the authors and do not necessarily represent those of their affiliated organizations, or those of the publisher, the editors and the reviewers. Any product that may be evaluated in this article, or claim that may be made by its manufacturer, is not guaranteed or endorsed by the publisher.
